# A Data-Based Approach for Selecting Pre- and Intra-Operative Language Mapping Tasks

**DOI:** 10.3389/fnins.2021.743402

**Published:** 2021-11-25

**Authors:** Justyna O. Ekert, Matthew A. Kirkman, Mohamed L. Seghier, David W. Green, Cathy J. Price

**Affiliations:** ^1^Wellcome Centre for Human Neuroimaging, UCL Queen Square Institute of Neurology, London, United Kingdom; ^2^Department of Neurosurgery, Queen’s Medical Centre, Nottingham, United Kingdom; ^3^Department of Biomedical Engineering, Khalifa University of Science and Technology, Abu Dhabi, United Arab Emirates; ^4^Department of Experimental Psychology, University College London, London, United Kingdom

**Keywords:** neurosurgery, fMRI, brain mapping, direct electrical stimulation (DES), language, object naming

## Abstract

**Background:** Pre- and intra-operative language mapping in neurosurgery patients frequently involves an object naming task. The choice of the optimal object naming paradigm remains challenging due to lack of normative data and standardization in mapping practices. The aim of this study was to identify object naming paradigms that robustly and consistently activate classical language regions and could therefore be used to improve the sensitivity of language mapping in brain tumor and epilepsy patients.

**Methods:** Functional magnetic resonance imaging (fMRI) data from two independent groups of healthy controls (total = 79) were used to generate threshold-weighted voxel-based consistency maps. This novel approach allowed us to compare inter-subject consistency of activation for naming single objects in the visual and auditory modality and naming two objects in a phrase or a sentence.

**Results:** We found that the consistency of activation in language regions was greater for naming two objects per picture than one object per picture, even when controlling for the number of names produced in 5 s.

**Conclusion:** More consistent activation in language areas for naming two objects compared to one object suggests that two-object naming tasks may be more suitable for delimiting language eloquent regions with pre- and intra-operative language testing. More broadly, we propose that the functional specificity of brain mapping paradigms for a whole range of different linguistic and non-linguistic functions could be enhanced by referring to databased models of inter-subject consistency and variability in typical and atypical brain responses.

## Introduction

Awake craniotomy with intra-operative stimulation mapping is strongly advocated for patients with gliomas affecting eloquent brain regions (Hamer et al., 2012; Leon-Rojas et al., 2020). A growing body of evidence suggests that more extensive resection is associated with longer survival (Sanai and Berger, 2008a; Sanai et al., 2008; McGirt et al., 2009; Ius et al., 2012; Jakola et al., 2012, 2017). Nevertheless, to preserve the patient’s quality of life, the survival benefit conferred by more aggressive surgery needs to be balanced with the risk of post-operative deficits (referred to as the onco-functional balance) (Duffau et al., 2009). This is particularly challenging in patients with tumors in or adjacent to cortical language hubs, where resection may lead to life-changing impairments in communication skills (Jakola et al., 2011; Gabel et al., 2019). To attempt to preserve the integrity of language regions, intra-operative mapping with the use of direct electrical stimulation is performed (Ilmberger et al., 2008; De Witte and Mariën, 2013; Rofes et al., 2017b). The capacity to detect and evaluate function during surgery critically depends on the selection of sensitive and lesion-site specific testing paradigms that, at present, lack standardization (O’neill et al., 2020; Sefcikova et al., 2020; Young et al., 2021).

The purpose of this study is to demonstrate how functional consistency maps generated from large populations of neurotypical controls can be used to facilitate the selection of pre- and intra-operative language tasks that robustly and consistently activate core language areas. Below, we discuss the challenges related to current language mapping practices prior to illustrating how results from functional magnetic resonance imaging (fMRI) of neurotypical participants can be used to inform decision making.

### Pre-operative Planning With fMRI

The complexity and wide distribution of language networks make it extremely challenging to predict how resection will affect language function. This is further complicated by inter-individual structural and functional variability, commonly observed in healthy individuals (Fedorenko and Blank, 2020) and exacerbated following tumor-induced reorganization. A common solution is to investigate language function prior to surgery using fMRI (Castellano et al., 2017). This provides potentially valuable, patient-specific information about the location and function of cortical language regions that may be at risk of damage, thereby enabling more targeted surgical approaches and reducing the operative duration (Sanai et al., 2008). In 2017, the American Society of Functional Neuroradiology published a white paper proposing two sets of language paradigms that balance the clinical usefulness and ease of application (Black et al., 2017). The recommended fMRI tasks for pre-surgical language assessment in adult patients included: sentence completion, silent word generation, rhyming, object naming, and/or passive story listening. The extent to which these guidelines have been adopted is currently unknown (Benjamin et al., 2018).

The reliability of fMRI has been examined in a meta-analysis of studies comparing fMRI with direct electrical stimulation for language mapping. The authors found that the sensitivity of fMRI for detecting language areas ranged from 59–100%, with 0–97% specificity (Giussani et al., 2010). It is also important to acknowledge three limitations of fMRI. First, detection of fMRI activation in a cortical area does not mean that the region is critical for a certain function and cannot be resected without post-operative functional deficits (Duffau, 2005; Silva et al., 2018) because the function of the region may be subsumed by another neural region/system. Second, when fMRI activation is not observed, true activation may have been missed because the blood-oxygen-level-dependent (BOLD) signal was compromised by pathology-related disruptions to neurovascular coupling. Third, the absence of fMRI activation in a region of interest may be due to an unsuitable paradigm that does not elicit robust activation at the individual subject level (Mahdavi et al., 2015; Pak et al., 2017), either because the region of interest is not strongly engaged or because of inter-subject variability in the degree to which a region is engaged.

Understanding inter-subject variability in neurologically normal subjects is important because it can arise for multiple reasons, such as differences in the hemodynamic response, differences in task performance or differences in the neural systems used for the same task (Seghier and Price, 2018). In this context, the absence of activation in a single patient might still be within the normal range but be treated as dysfunctional if located in a region that is significantly activated in a group-level study.

Given the above, the results from fMRI analyses are not a substitute for intra-operative stimulation mapping; however, they are useful for helping to select tasks that are most likely to evoke a response during intra-operative stimulation mapping.

### Intraoperative Stimulation Mapping

The application of direct electrical stimulation (DES) for mapping of motor and sensory pathways in neuro-oncological surgery was described by Mitchel Berger in the nineties and it has subsequently become a part of the neurosurgeon’s armamentarium (Berger et al., 1989; Berger and Ojemann, 1992). DES is the gold standard used to map the function of eloquent cortical regions and subcortical white matter tracts in neurosurgery patients, thereby facilitating maximum safe resection (Hamer et al., 2012). For intra-operative language mapping, the patient must be awakened or remain awake throughout the surgery so that they can engage in linguistic tasks such as object naming, counting, verbal fluency, and other (for review, see Young et al., 2021). A neuropsychologist or speech and language therapist monitors the patient’s response to the task while the peritumoral tissue is stimulated to transiently disrupt its function (Klitsinikos et al., 2021). This involves bipolar stimulation with progressively increasing current intensity, typically from 1.5 to 6 mA and maintaining contact with neural tissue for 3 s at a time (Sanai et al., 2008). According to the standard protocol first established by Ojemann et al. (1989), each brain region should be stimulated at least 3 times (Sanai and Berger, 2008b; Sanai et al., 2008; Hervey-Jumper et al., 2015). A positive language site is identified when stimulation to the cortical region of interest results in an inability to successfully perform the task in 66% or more of the testing (Sanai and Berger, 2010).

Functional disturbances during electrical stimulation may indicate that the stimulated region was required for the task tested, but they may also reflect false positives. For example, a reduction in speed or accuracy may not be due to disturbance at the stimulus site; it could be a consequence of (a) disruption in distant task-related regions through the spread of electrical current along connecting axons (Matsumoto et al., 2004; Mandonnet et al., 2010), (b) patient fatigue, particularly during long testing sessions (Mandonnet et al., 2010), or (c) inadequate task difficulty (Bu et al., 2021). Conversely, there are several reasons why the absence of an effect of DES may be a false negative: (1) the task was not appropriate to test the function of the stimulated region because it does not activate the region in the normal population; (2) the stimulated region was essential for the task in the patient because of normal inter-subject variability or pathology-induced functional reorganization; and (3) the stimulated region is required for the task but the stimulation intensity was insufficient to generate a response or the effect wasn’t detected, e.g., if the effect was on response times or hesitation rather than speech arrest (Shimotake et al., 2015; O’neill et al., 2020). The successful interpretation of intra-operative DES is therefore critically dependent on selecting tasks that: (i) are easy to perform, particularly for patients who struggle to maintain focus during awake surgery; and (ii) robustly and consistently activate the targeted region in neurotypical individuals within the short timespan that DES can be safely applied.

At present, no standardized protocol exists to reliably identify and test language regions in neurosurgery patients with many institutions assessing only one task (Ruis, 2018; Sefcikova et al., 2020). A survey of the European Low-Grade Glioma Network showed that object naming was the most frequently utilized task for mapping language during awake surgery (Rofes et al., 2017a). However, choice of the object naming paradigms is highly variable across institutions ranging from in-house designed paradigms to use of one of a number of standardized tests for intra-operative language assessments, such as DO70/DO80, Picture Naming AAT, Boston Naming Test, Reitan Indiana Aphasia screening test, BDAE and the Snodgrass and Vanderwart collection, Laiacona–Capitani test (Rofes et al., 2015; Ruis, 2018; O’neill et al., 2020).

While stimulation mapping with visual picture naming is considered the gold standard, the choice of stimulus modality should be carefully considered, taking into account the site of the lesion. Hamberger et al. (2005) showed that sparing visual naming sites, without consideration of other sites, did not reliably prevent post-operative language decline in patients with temporal lobe epilepsy. Six out of seven patients who had auditory naming sites resected declined post-operatively, in comparison to three out of twelve patients with preserved auditory naming regions. Intra-operative language mapping may therefore require multiple tasks in order to prevent post-operative language deficits (Manan et al., 2020).

### Current Study

The current study investigates how robustly and consistently different object naming paradigms engage sensory, motor and language regions in neurotypical individuals. As a proxy for neural activity, we used BOLD fMRI. By identifying object naming paradigms with the most consistent and robust BOLD responses, we generate hypotheses for optimal task selection for intra-operative and pre-operative surgical planning.

**FIGURE 1:**
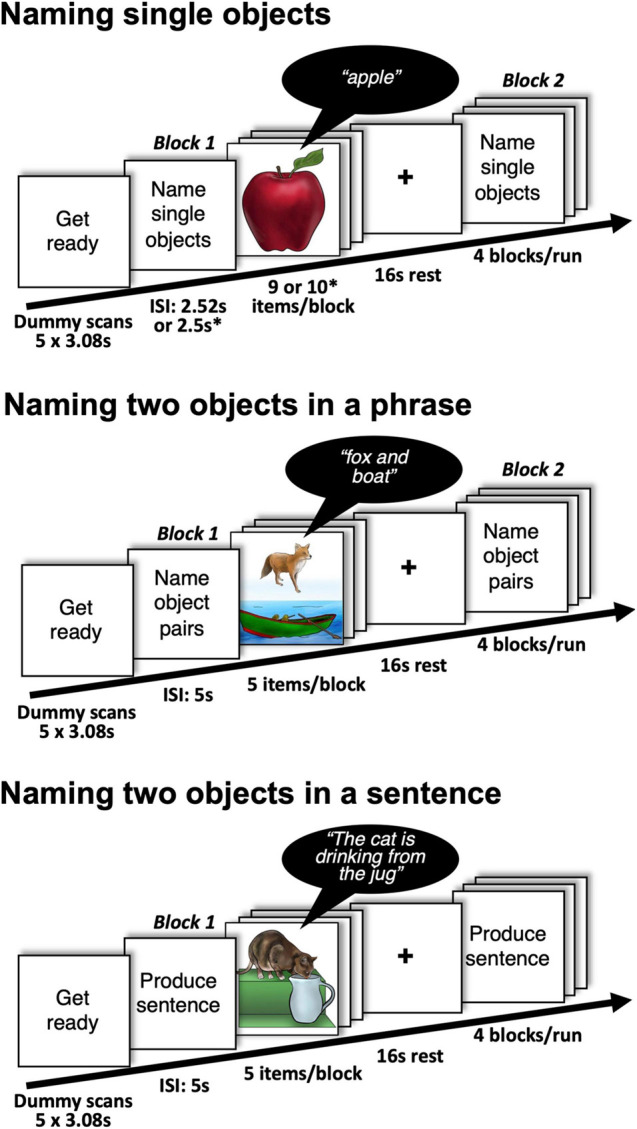
Details of the experimental design with examples of stimuli and expected responses during naming single and two objects. Participants were also asked to perform a single auditory object naming task (naming objects from sounds). ^∗^presentation parameters used in Group 2.

Specifically, we compared how consistently four different object naming tasks activated sensory, motor and language regions in neurotypical individuals at the voxel/region level. Three of the object naming tasks involved visual (picture) naming, the third involved auditory object naming (from the non-verbal sounds of objects and animals). For all four tasks, the number of stimuli presented and the fMRI acquisition time was controlled but, for two of the visual naming tasks, we presented objects in pairs every 5 s for a duration of 2.5 s, whereas in the other two tasks we presented one object at a time every 2.5 s for a duration of 1.5 s (Figure 1).

We expected that the requirement to name two objects on a trial, rather than one, would increase demand on the regions involved in speech production (e.g., those required to retrieve and produce names) and so yield more robust activation at the individual level. If the naming of two objects results in more consistent activation at the individual level in speech production regions compared to naming a single object, then future studies could investigate whether naming two objects increases test sensitivity for intraoperative and pre-operative language mapping.

Prior studies have aimed to compare the effectiveness of different language mapping fMRI paradigms (e.g., Unadkat et al., 2019) using traditional SPM{t} maps. However, this approach does not account for inter-subject variability (or consistency) and relies on selecting an arbitrary t-score threshold, leading to possible bias. In contrast, our functional consistency maps can be used to visualize activation over a range of different statistical thresholds and provide a score to indicate how consistently activation is observed across subjects in each voxel (Seghier and Price, 2016).

We used a large heterogeneous sample to deliberately maximize inter-subject variability (e.g., in age and gender). This heterogeneity makes our results more generalizable to clinical populations. As inter-subject variability in sample demographics was held constant across tasks, any task dependent differences in the degree to which a brain region is activated cannot be attributed to selection bias. Our goal was to identify tasks that result in the most consistent fMRI responses across participants, despite the heterogeneity in the subject characteristics. For tasks with low consistency (i.e., high inter-subject variability) in fMRI activation across the whole sample, we investigated whether consistency differed for younger compared to older participants.

## Materials and Methods

The data used in this experiment were selected from the PLORAS database (Seghier et al., 2016) rather than being acquired specifically for the purposes of the current experiment. Data collection was approved by the London Queen Square Research Ethics Committee. All subjects gave written informed consent prior to scanning.

### Participant Groups

Our participants included 79 native English speakers, with normal or corrected-to-normal vision, and no history of neurological or psychiatric disorders. All were right handed according to the Edinburgh Handedness Inventory (Oldfield, 1971).

The 79 participants comprised two groups (Group 1 and Group 2). Group 1 (*n* = 24) performed two different object naming tasks, counterbalanced across participants. The first object naming task (single visual object naming) involved overtly naming a single object in a picture (see Figure 1). Successive objects were semantically unrelated. The second object naming task (single auditory object naming) involved hearing the sound of an object or animal (e.g., a guitar playing) and overtly naming the object associated with the sound (e.g., “*guitar*”). Group 2 (*n* = 55) performed 4 object naming tasks including those performed by Group 1 (single visual object naming and single auditory object naming) and two tasks that presented two semantically unrelated objects per picture (see Figure 1). In one task, the objects were juxtaposed one above the other and participants named both objects aloud one after the other using a noun phrase (e.g., “*fox and boat*”). In the other task, the objects interacted to depict an event and participants were instructed to overtly name the two objects within a sentence that described how the objects were interacting (e.g., “*The cat is drinking from the jug*”). To do so they used one of four pre-specified verbs that described the interaction: “eating,” “drinking,” “jumping,” or “falling.” The set of acceptable verbs was restricted to minimize inter-subject variability in verb selection. Passive constructions were ruled out by requiring the agent of the action to be named first. We expected inter-subject consistency to be highest for sentence production because this is the most challenging task and therefore most demanding on the language system.

### Other fMRI Tasks for Group 1

Group 1 participated in 16 different tasks including the visual and auditory single object naming tasks that we focus on in the current paper. In brief, the 16 tasks comprised a 2 × 2 × 2 × 2 factorial design (Hope et al., 2014). Factor 1 was visual or auditory stimuli, factor 2 was semantic content (stimuli were either meaningful or meaningless), factor 3 was phonological content (stimuli were either verbal or non-verbal) and factor 4 was task (either speech production or 1-back matching). The object naming tasks are examples of “non-verbal semantic stimuli.” The verbal semantic stimuli were written or heard object names. The verbal non-semantic stimuli were written or heard pseudowords (e.g., “*wrundle*”). The non-verbal non-semantic stimuli were colored patterns in the visual modality and meaningless humming in the auditory modality. During speech production, participants: named the objects, read or repeated the words and pseudowords, named the color of the meaningless visual stimuli or named the gender of the humming voices in the auditory modality. During 1-back matching, participants indicated whether the stimulus was identical or different to the preceding stimulus. For each subject, the stimuli presented during speech production were identical to the stimuli presented during 1-back matching.

The order of the 16 tasks was counterbalanced across 24 subjects. Half the subjects performed the speech production tasks first and half performed the 1-back matching tasks first. Within each of these groups, half were presented the visual stimuli first, then auditory stimuli, the other half were presented with auditory stimuli first then visual stimuli. Within each of these groups, each type of stimulus (words, pseudowords, objects, colored patterns/humming) occurred an equal number of times first, second, third, or fourth (across subjects). As exactly the same stimuli were used for speech production and 1-back matching tasks, a direct comparison of fMRI activation for speech production and 1-back matching identified brain regions involved in speech production, after controlling for stimuli. The main effects of stimulus modality (visual versus auditory), semantics versus non-semantic, and phonological versus non-phonological are reported in Hope et al. (2014). The current study examines inter-subject consistency across the whole brain, and in language regions that are (a) activated by object naming compared to rest and (b) also activated when retrieving speech sounds, after controlling for task and perceptual processing (see “Regions of interest” below for details).

### Other fMRI Tasks for Group 2

**FIGURE 2:**
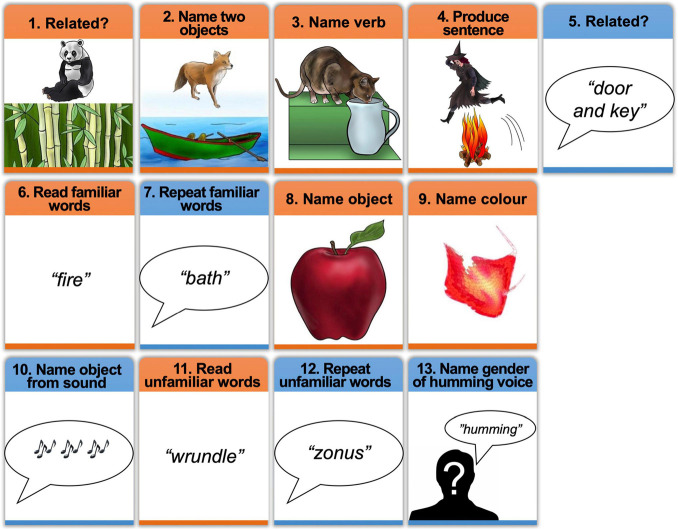
Schematic of the experimental tasks with examples of stimuli. The order of tasks (1–13) was the same for all participants in Group 2. Tasks 6–13 were also presented to Group 1, in counterbalanced order. Orange boxes = tasks in the visual modality, Blue boxes = tasks in the auditory modality. In task 10 (naming object from sound), participants heard a sound (e.g., a guitar playing) and were asked to name the object that produced the sound (e.g., “guitar”).

Group 2 participated in 13 different tasks (see Figure 2) including the 4 object naming tasks described above. The 13 tasks comprised 2 experiments. The first experiment involved 5 tasks that each presented two objects in a trial and were always presented in the following order: (1) visual semantic matching, (2) naming two objects, (3) verb naming, (4) sentence production and (5) auditory semantic matching. Tasks 1–4 presented two objects in each picture. In the visual semantic matching task, half the stimuli presented pairs of semantically related objects, and half presented pairs of objects that were not semantically related, with participants indicating this relationship with one finger press for semantically related and another for semantically unrelated. In the two-object naming task, the two objects in the picture were unrelated and non-interacting (e.g., “fox and boat”), see Figures 1, 2. In the verb and sentence production tasks, the two objects in the picture were interacting and participants, either produced the verb describing the interaction (“Drinking”) or generated a short sentence (as described above and in Figures 1, 2). In auditory semantic matching, participants heard two object names that were either semantically related or not, indicating this relationship with a finger press response. Further details about the 5 tasks in Experiment 1 have been reported in Sanjuán et al. (2015). Here we focus on naming two objects in a phrase or in a sentence.

The second experiment in which Group 2 participated involved the 8 speech production tasks used with Group 1 (tasks 6–13 in Figure 2), including visual and auditory single object naming. These 8 tasks were always performed after the 5 Experiment 1 tasks. As in Experiment 1, the order of the 8 tasks in Experiment 2 was held constant. Moreover, the stimuli used in each task (Experiment 1 and 2) were identical for every subject in Group 2. This was to ensure that inter-subject variability, within task, could not be accounted for by stimulus effects. However, as Group 2 always performed Experiment 2 after Experiment 1, differences between tasks (e.g., naming two objects per trial in Experiment 1 vs. naming a single object per trial in Experiment 2) could reflect task order, see section on investigating the effect of task order below.

### Stimulus Creation

The same selection of stimuli was presented to Groups 1 and 2. Stimulus creation was initiated by identifying 128 objects and animals with highly familiar names. Each was drawn and colored as realistically as possible by a professional artist (Eldad Druks). Edges and features were outlined in black to ensure that the objects were easily recognizable in the scanner (see Figure 1), confirmed by high naming accuracy in pilot studies. The sounds of objects were taken from the NESSTI sound library (Hocking et al., 2013) but only 32 of the 128 objects in the pictures had sounds that were unambiguously related to one object or animal (e.g., there is no sound associated with a banana or table). Words were the written or spoken names associated with the objects. Pseudowords were created using a non-word generator (Duyck et al., 2004) that matched written pseudowords to the 128 objects names for bigram frequency, number of orthographic neighbors and word length. The colored patterns were created from the object pictures by scrambling the global and local features to render them unrecognizable and then manually editing the images to accentuate one of eight colors (brown, blue, orange, red, yellow, pink, purple, and green). We selected 32 different visual forms/patterns, with 4 shades of each of the 8 color categories (i.e., 8 different naming responses). In the auditory non-semantic-non-phonological task, there were 32 different humming sounds but only two possible responses (male/female).

The colors were not uniform in either the object naming or color naming tasks but pilot studies ensured that participants agreed on the predominant color of all the visual patterns. The stimuli used in the gender naming task (meaningless humming) were created by male or female voices humming with no phonological or semantic content.

### Counterbalancing Objects Across Tasks

For Group 1, the 128 object names were assigned to four different sets of 32 stimuli (A, B, C, and D). Each set also included 4 repeat stimuli that needed to be detected during 1-back matching (i.e., total number of stimuli = 36). Sets A–C were rotated across pictures of objects, written object names and auditory object names, in different participants. Semantic and phonological content was therefore controlled across participants. Within participant, no stimulus set was repeated across the speech production tasks or across the 1-back matching tasks. Set D included the sounds of 32 objects that were always used during the object sound tasks and never used in any other task.

For Group 2, the 120 stimuli were assigned to 6 different sets of 20 stimuli (A-F), with 8 stimuli in set G. Each task, except auditory object naming, presented 2 different stimulus sets. In Experiment 1, the first task presented two novel sets (A and C), and the second to fifth presented one novel set (not presented in a previous task) and one repeated set (E and A, B and C, F and E, and D and F for tasks 2–5). In Experiment 2, visual object naming presented sets D and F. The pictures in set D were novel but their names had been presented during auditory semantic matching in Experiment 1. The pictures in set F were not novel as they had previously been presented for sentence production. For auditory object naming, participants were presented with 8 new stimuli from set G and 12 stimuli that had previously been seen or heard in Sets A to E.

### Investigating the Effect of Stimulus Familiarity

As described above, the stimuli presented to Group 2 during two-object naming and sentence production (Experiment 1) were less familiar than the stimuli presented to Group 2 during auditory and visual single object naming (Experiment 2). Many prior studies have demonstrated how stimulus familiarity reduces neuronal responses, see Van Turennout et al. (2003) for an illustration during object naming. If the neuronal response is reduced by stimulus repetition, sensitivity to fMRI changes may be reduced possibly leading to less consistency in activation across subjects. To investigate the effect of familiarity, we compared inter-subject variability for single object naming in (A) Group 1 versus Group 2 and (B) subjects in Group 1 who performed speech production before (*n* = 12) vs. after (*n* = 12) 1-back matching. For (A), the names of objects in the object naming tasks were completely novel for Group 1 but not for Group 2 (see above). For (B) the pictures of objects in the object naming tasks were completely novel for the 12 subjects who performed the speech production tasks first, but not novel for the 12 subjects who performed 1-back matching first.

### Presentation Details

Each task (16 for Group 1 and 13 for Group 2) was presented in its own (separate) scanning run with 4 blocks of stimuli, each lasting 25 s, followed by 16 s of fixation. Within block, there were 9 stimuli of the same kind (8 novel, 1 repeat) for all Group 1 tasks; and 10 stimuli for all Group 2 tasks. The stimulus repeat in the Group 1 tasks only needed to be detected and responded to (with a finger press) in the 1-back matching tasks but was also present in the speech production tasks in order to keep the stimuli constant across tasks. The inter-stimulus interval was 2.52 s for Group 1, 2.5 s for Group 2 Experiment 2, and 5 s for Group 2 Experiment 1 (which presented pairs of object stimuli), see Table 1 for further details of stimulus presentation parameters).

**TABLE 1:** Experimental details for Group 1 and Group 2.

	**Group 1**	**Group 2**
**Participants**		
Number	25	59
Gender (n females/n males)	12/12	34/25
Mean age in years (+/-SD)	31.44 (5.74)	44.5 (17.66)
**Average number of syllables (SD)**		
Reading words	1.53 (0.68)	1.55 (0.68)
Repeating words	1.53 (0.68)	1.68 (0.73)
Reading pseudowords	1.94 (0.92)	1.50 (0.51)
Repeating pseudowords	1.90 (0.84)	1.50 (0.51)
Naming pictures	1.55 (0.69)	1.48 (0.72)
Naming sounds	1.81 (0.92)	1.88 (0.94)
Naming gender	1.50 (0.51)	1.50 (0.51)
Naming colors	1.36 (0.49)	1.40 (0.50)
**Average number of letters (+/-SD)**		
Reading words	5.24 (1.68)	5.08 (1.61)
Repeating words	5.24 (1.68)	5.28 (1.38)
Reading pseudowords	5.28 (1.94)	4.40 (1.03)
Repeating pseudowords	5.35 (1.72)	4.35 (1.08)
Naming pictures	5.30 (1.75)	5.28 (1.75)
Naming sounds	5.64 (2.21)	5.65 (2.40)
Naming gender	5.00 (1.01)	5.00 (1.01)
Naming colors	4.89 (1.04)	4.80 (1.18)
**Stimulus duration in sec (+/-SD)**		
Sentence production	N/A	2.5
Two-object naming	N/A	2.5
Single visual object naming	1.5	1.5
Single auditory object naming	1.47 (0.12)	1.45 (0.15)
**Inter-stimulus interval in sec**		
Sentence production	N/A	5.0
Two-object naming	N/A	5.0
Single visual object naming	2.52	2.5
Single auditory object naming	2.52	2.5
**Block length in sec**		
Sentence production	N/A	25
Paired object naming	N/A	25
Single visual object naming	25.2	25
Single auditory object naming	25.2	25
**Scanning parameters**		
TR (sec)	3.085	3.085
Number of slices per image	44	44

### Procedure

Prior to scanning, we trained each participant on all tasks using a separate set of training stimuli except for the environmental sounds which remained the same. When in the scanner, participants were instructed to respond as fast as possible, keeping their body and head as still as possible and their eyes open and fixated on a cross in the middle of the display screen. Scanning started with the instructions “Get Ready” written on the in-scanner screen while five dummy scans were acquired (15.4 s in total). This was followed by a written instruction (e.g., “Name”), lasting 3.085 s, which indicated the forthcoming start of a new block and reminded participants of the task that needed to be performed.

Auditory stimuli were presented via MRI compatible headphones (MR Confon, Magdeburg, Germany), which filtered ambient in-scanner noise. Volume levels were adjusted for each participant before scanning. Spoken responses were recorded via a noise-canceling MRI microphone (FOMRI IIITM Optoacoustics, Or-Yehuda, Israel), and transcribed manually for off-line analysis. Correct responses were those that matched the target without delay or self-correction. For two-object naming and sentence production, the response was only correct if both objects were named correctly within the inter-trial interval. In addition, for sentence production, the correct verb also needed to be produced. For some stimuli, more than one response was considered correct. For example, a picture of a mug could be named “cup” or “mug.” All other responses were categorized as incorrect. Response times for speech production were analyzed off-line but were only available for Group 2.

### fMRI Data Analysis

Data for Group 1 and 2 were processed independently. All preprocessing and statistical analysis were performed in SPM12 (Wellcome Trust Centre for Neuroimaging, University College London, United Kingdom), running on MATLAB 2012a (Mathworks, MA, United States). Functional volumes were spatially realigned to the first EPI volume and unwarped to compensate for non-linear distortions caused by head movement or magnetic field inhomogeneity. The unwarping procedure was used in preference to including the realignment parameters as linear regressors in the first-level analysis because unwarping accounts for non-linear movement effects by modeling the interaction between movement and any inhomogeneity in the T2^∗^ signal. After realignment and unwarping, the realignment parameters were checked to ensure that participants moved less than one voxel (3 mm) within each scanning run. The anatomical T1w images were co-registered to the mean EPI image generated during the realignment step and then spatially normalized to the MNI space using the unified normalization-segmentation routine in SPM12.

To spatially normalize all EPI scans to MNI space, the deformation field parameters that were obtained during the normalization of the anatomical T1w image were applied. The original resolution of the different images was maintained during normalization (voxel size 1 × 1 × 1 mm^3^ for structural T1w and 3 × 3 × 3 mm^3^ for EPI images). After normalization, functional images were spatially smoothed with a 6 mm full-width-half-maximum isotropic Gaussian Kernel to compensate for residual anatomical variability and to permit application of Gaussian random-field theory for statistical inference (Friston et al., 1995).

### First Level Statistical Analyses

All preprocessed functional volumes were entered into a subject specific fixed effect analysis using the general linear model. Stimulus onset times were modeled as single events. For Group 1, we used 2 regressors per task, one modeling instructions, and the other modeling each stimulus. For Group 2, we used 4 regressors per task to model: (i) instructions, (ii) stimuli with correct responses, (iii) stimuli with incorrect responses and (iv) “other” responses (delayed, no response, or self-corrected). Stimulus functions were convolved with a canonical hemodynamic response function and high pass filtered with a cut-off period of 128 s.

For each scanning session/run (that alternated one task of interest with fixation), we generated a single contrast that compared activation in response to the stimuli and task of interest to resting with fixation. This resulted in 16 different contrasts (one per task) for each participant for Group 1 and 13 different contrasts for Group 2. Visual inspection ensured that there were no visible artifacts (e.g., edge effects, activation in ventricles) that might have been caused by within-scan head movements. These contrast images were then entered into a second-level analysis in SPM12 so that we could functionally segregate our core regions of interest.

### Inter-Subject Consistency During Object Naming

Inter-subject consistency for all object naming tasks was evaluated, at every brain voxel, using threshold-weighted voxel-based consistency maps, as described in Seghier and Price (2016). These “functional consistency maps” quantify the proportion of subjects activating a particular voxel, and its nearest 6 neighbors, over a wide range of statistical thresholds (*p* < 0.5–0.001). Threshold-weighted consistency maps are generated by defining a complementary cumulative histogram of the number of subjects against the statistical threshold *th* at each voxel. Consistency is expressed as a single number by calculating the area under the curve of the complementary cumulative histogram. Prior to estimating the area under the curve, the generated histograms were multiplied by a linear weighting function *W*_*th*_ that monotonically increased with *th*:


$$W_{t\,h}=\frac{2}{T_{m\,a\,x}-T_{m\,i\,n}}\times t\,h$$

The histograms were thus linearly weighted to assign more weight to individual effects at higher statistical thresholds. The minimum threshold *T*_*min*_ was set to *p* = 0.5 (uncorrected) to exclude effects of non-interest. To account for the spatial dependency between neighboring voxels, the voxel-based consistency value summarized the effect at the voxel of interest and its 6 nearest neighbors using a spherical volume of interest with radius of 2 mm. A low consistency value (the proportion near 0) means that the voxel was consistently not activated in almost all subjects. When the proportion is 1, the voxel was activated in each subject irrespective of threshold within the range of statistical thresholds. A proportion less than 1, indicates either consistency across subjects at a low statistical threshold or that only a subset of participants activated the voxel, irrespective of threshold (for full discussion about the interpretation of intermediate consistency values, see Seghier and Price, 2016).

The “functional consistency maps” generated for each of our naming tasks allowed us to compare inter-subject consistency in activation, within language regions of interest (see below) and across the whole brain, for different tasks.

To investigate whether inter-subject variability is greater in older than younger participants, we split our 79 participants into two approximately equal sized groups (40 under 35 years old vs. 39 over 35 years old) and compared consistency across these groups for naming one object in (i) the visual modality and (ii) the auditory modality. In addition, we split the 55 participants who named two objects per condition and produced sentences into two approximately equal sized groups (27 under 40 years old versus 28 older than 40 years old) and compared consistency across age groups for the same tasks.

### Regions of Interest

In addition to considering inter-subject consistency in activation at the whole brain level, we also home in on the core language areas that are involved in extracting and producing speech sounds, specifically the left posterior superior temporal cortex (Wernicke’s area) and the left inferior frontal cortex (Broca’s area). Using data from Group 1 only, these regions were segregated from the rest of the object naming network by searching for voxels that were activated during (A) object naming compared to rest and (B) 1-back matching of written words and pseudowords compared to object and color naming. Contrast (B) has already been shown to activate areas involved in speech sound processing (Hope et al., 2014), consistent with expectation that skilled readers are highly trained to link written words and pseudowords to speech sounds and these “phonological codes” can be used to make 1-back matching decisions. Common activation for (Contrast A) and (Contrast B) segregates speech sound processing from the rest of the object naming system because (i) the 1-back matching task does not involve motor control of speech or auditory processing of the spoken response; and (ii) areas involved in visual perception are controlled by comparing visual 1-back matching of written words and pseudowords to visual objects and colored patterns.

Common activation for Contrasts (A) and (B) was identified by using a global conjunction in SPM with a statistical threshold of *p* < 0.05 after family wise error correction for multiple comparisons across the whole brain (in height). In addition, we checked and confirmed that the identified voxels were also activated by 1-back matching of words and pseudowords compared to rest.

**FIGURE 3:**
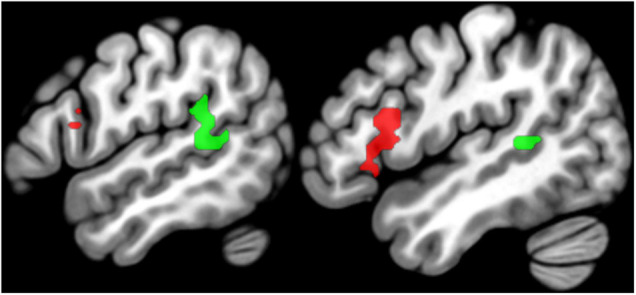
Regions of interest. Sagittal slices (left x = –54, right x = –48) showing the group-level SPM{t} map for language regions of interest overlaid on a standard structural template in MNI space at *p* < 0.05 corrected for multiple comparisons. The SPM{t} map was generated using data from Group 1 only. Green = temporal regions of interest, Red = frontal regions of interest (see Table 2 for details).

The left temporal and frontal regions activated by the conjunction are illustrated in Figure 3 and Table 2. The left frontal region included the pars opercularis (pOp) and pars triangularis (pTri). The left temporal region was in the left anterior ascending terminal branch of the superior temporal sulcus (atSTS), extending posteriorly into the left middle temporal gyrus (MTG) and dorsally into the left temporo-parietal junction (TPJ).

**TABLE 2:** Statistical details for regions of interest.

**Region**	**Anatomical label (abbreviation)**	**MNI co-ordinates**	**Vx**	**Z-scores**		
				**Visual**		
				**SP**	**1-b**	**Conj.**
				**O > rest**	**W and P > O and C**	**(SP and 1-b)**
Frontal	Pars triangularis (pTri)	−42, 30, −3	161	5.89	4.27	7.47
		−45, 24, 6		5.73	3.53	6.42
	Pars opercularis (pOp)	−48, 15, 18		5.86	3.67	6.63
Temporal	Anterior ascending terminal branch of the STS (atSTS)	−51, −45, 6	69	3.61	3.71	6.06
	Middle temporal gyrus (MTG)	−60, −48, 9		2.55	3.65	5.80
	Temporo-parietal junction (LTJ)	−54, −42, 21		5.41	3.09	5.76

*SP = speech production, 1-b = 1-back matching. W = written words; P = written pseudowords; O = objects in pictures; C = colored patterns; Vx = number of voxels activated. Conj. = conjunction of SP O > rest and 1-b W and P > O and B.*

## Results

### Behavioral Data

**FIGURE 4:**
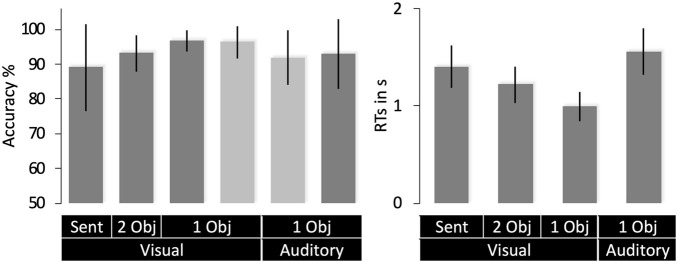
Behavioral data (mean with SD). Accuracy scores for Group 1 are shown in light gray and Group 2 in dark gray. RTs (right) were only available for Group 1. 1 Obj = single object naming, 2 Obj = two-object naming, Sent = sentence production. RTs are for correct trials only.

Average in-scanner accuracy was 89% or above for each object naming task in both groups (Figure 4). Response times were only available for Group 2. Within this group, response times were slower for single object naming in the auditory than visual modality (Figure 4) because auditory stimuli were delivered over time (sequential) while all parts of the visual stimuli were presented at the same time point (simultaneous).

### Inter-Subject Consistency in Object Naming Activation

**FIGURE 5:**
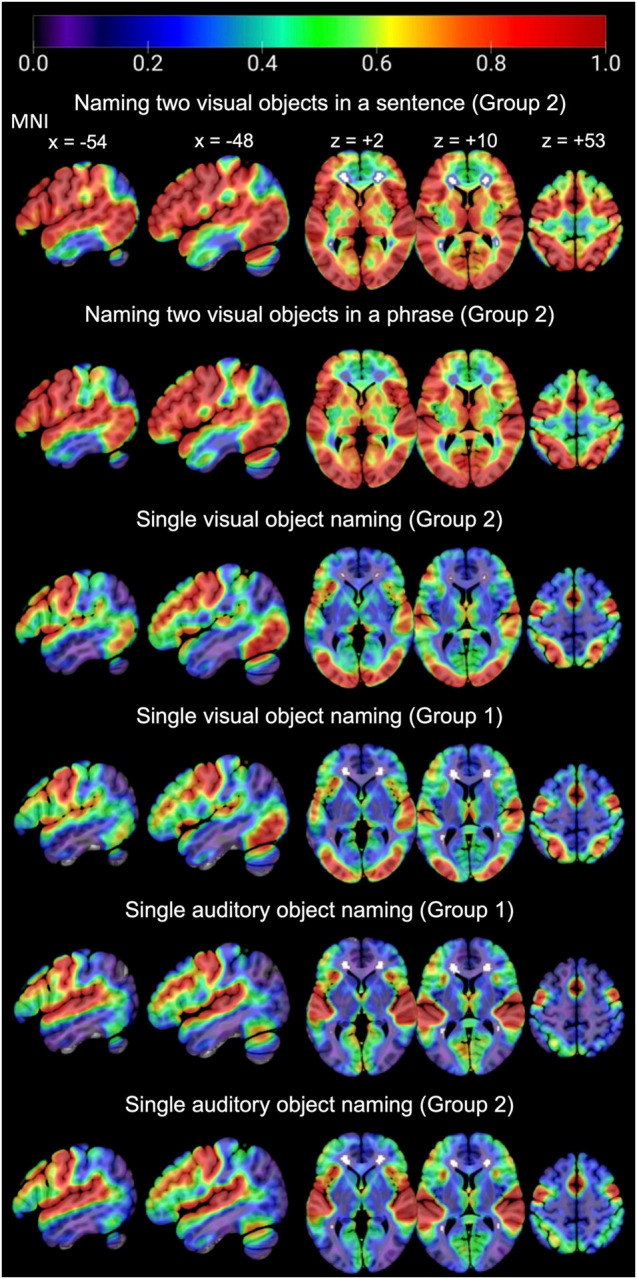
Consistency of activation across the whole brain, for each task of interest in each group.

For naming single objects and two objects, activation was highly consistent in sensori-motor areas, including bilateral occipital, motor, and auditory cortices (see Figure 5). These regions were associated with the following functions in the group-level analysis reported by Hope et al. (2014): (i) bilateral occipito-temporal regions were associated with visual perception, (ii) left posterior middle temporal and parietal areas were associated with semantic associations, (iii) bilateral motor cortices, supplementary motor cortices, subcortical and cerebellar regions were associated with motor control of speech; and (iv) bilateral auditory cortices were associated with hearing stimuli or hearing the sound of the spoken response.

**FIGURE 6:**
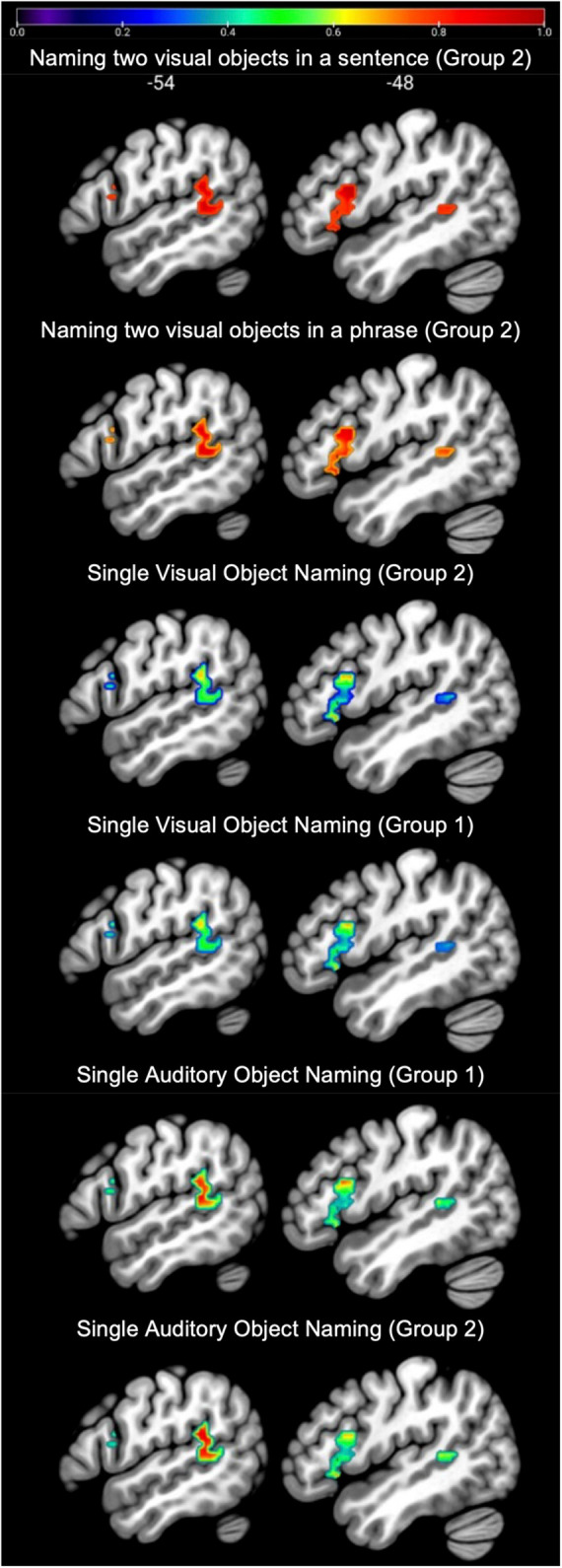
Consistency in activation within language regions of interest.

In language regions of interest (left posterior superior temporal and inferior frontal regions associated with retrieving speech sounds, see Figure 3), activation was also highly consistent for naming two objects in a noun phrase and for naming two objects in a sentence (Group 2) but significantly less consistent for naming single visual objects in Groups 1 and 2, see Table 3 and Figure 6 for details. Auditory single object naming was significantly more consistent than visual single word object naming in temporal regions but not in frontal regions (see Table 3C).

**TABLE 3A:** Consistency of activation in regions of interest.

**Region**	**MNI co-ordinates**	**Naming objects**					
		**Two in a sentence**	**Two in a phrase**	**Single**			
		
		**Visual**				**Auditory**	
		**G2**	**G2**	**G1**	**G2**	**G1**	**G2**
pTri	−42, 30, −3	90%	89%	59%	59%	64%	58%
	−45, 24, 6	84%	84%	53%	50%	55%	50%
pOp	−48, 15, 18	93%	85%	53%	55%	60%	62%
atSTS	−51, −45, 6	87%	87%	42%	47%	57%	69%
MTG	−60, −48, 9	90%	84%	42%	53%	67%	80%
TPJ	−54, −42, 21	92%	88%	43%	65%	75%	87%

**TABLE 3B:** Statistical comparison of naming one or two objects per trial.

**Region**	**MNI co-ordinates**	**Odds ratios for naming two**				
		**objects in a phrase compared to**				
		
		**Sentence**	**Single object naming**			
		**Visual**			**Auditory**	
		**G2**	**G1**	**G2**	**G1**	**G2**
pTri	−42, 30, −3	0.93*	5.87	5.44	4.67	5.87
	−45, 24, 6	0.98*	4.58	5.11	4.26	4.93
pOp	−48, 15, 18	0.41*	5.27	4.90	3.92	3.63
atSTS	−51, −45, 6	0.99*	9.54	7.65	4.93	3.07*
MTG	−60, −48, 9	0.57*	7.11	4.58	2.49*	1.28*
TPJ	−54, −42, 21	0.64*	8.86	3.62	2.34*	1.00*

**TABLE 3C:** Statistical comparison of auditory and visual single object naming.

**Region**	**MNI co-ordinates**	**Odds ratios for auditory compared**			
		**to visual single object naming**			
		
		**Auditory G1 vs**		**Auditory G2 vs**	
		**Visual G1**	**Visual G2**	**Visual G1**	**Visual G2**
pTri	−42, 30, −3	1.26*	1.17*	1.00*	0.93*
	−45, 24, 6	1.08*	1.20*	0.93*	1.04*
pOp	−48, 15, 18	1.34*	1.25*	1.45*	1.35*
atSTS	−51, −45, 6	1.94*	1.55*	3.11	2.49
MTG	−60, −48, 9	2.86	1.84*	5.57	3.59
TPJ	−54, −42, 21	3.78	1.55*	8.86	3.62

*All odds ratios, except the values indicated with an asterisk, were significant (*p* < 0.05, 2 tailed), using both Chi Squared (Pearson) *p* values and Fischer’s exact probability test. Consistency is expressed as a percentage (between 0–100%) rather than a value between 0 and 1. G1 = Group 1; G2 = Group 2. atSTS = anterior ascending terminal branch of the superior temporal sulcus, MTG = middle temporal gyrus, TPJ = temporo-parietal junction, pOp = pars opercularis, pTri = pars triangularis.*

Despite our expectation that sentence production would produce more consistent activation than two-object naming, there was no significant difference in the consistency of activation for producing two object names in a phrase compared to in a sentence (see Table 3B). We also found no significant difference in single object naming between (A) Group 1 (less familiar names) and Group 2 (more familiar names); or (B) subjects in Group 1 who performed speech production tasks before versus after 1-back matching on the same stimuli (mean consistency = 52% for both subgroups). Therefore, there was no evidence that differences in activation consistency for naming two objects rather than a single object arose from either condition order, stimulus familiarity or fatigue.

A comparison of inter-subject consistency for older and younger participants revealed remarkable similarity across older and younger participants for the single object naming tasks (see Table 4 for details). For naming two objects in phrases and sentences, inter-patient consistency appeared higher for older than younger participants. However, this was neither anticipated nor significant using two-tailed Fisher’s exact test. In summary, inter-patient consistency was significantly different between tasks for each age group, but not significant between age groups (for any of the tasks).

**TABLE 4:** Consistency of activation for younger versus older participants.

**Region**	**MNI co-ordinates**	**Naming objects**							
		
		**Two in a sentence**		**Two in a phrase**		**Single**			
			
		**Visual**				**Visual**		**Auditory**	
			
		**Young**	**Older**	**Young**	**Older**	**Young**	**Older**	**Young**	**Older**
		***n* = 27**	***n* = 28**	***n* = 27**	***n* = 28**	***n* = 40**	***n* = 39**	***n* = 40**	***n* = 39**
pTri	−42, 30, −3	84%	95%	80%	93%	59%	56%	56%	60%
	−45, 24, 6	80%	88%	84%	82%	56%	47%	51%	52%
pOp	−48, 15, 18	91%	96%	80%	93%	56%	59%	56%	70%
atSTS	−51, −45, 6	79%	95%	78%	93%	44%	48%	67%	64%
MTG	−60, −48, 9	87%	93%	80%	87%	52%	51%	79%	76%
TPJ	−54, −42, 21	87%	96%	82%	94%	65%	63%	86%	81%

*For abbreviations, see Table 3.*

## Discussion

To select the optimal task for intra-operative mapping, a neurosurgeon needs confidence that the selected task typically and robustly engages the function of interest. In the case of object naming, our results strongly favor use of a two-object naming paradigm compared to a single object naming paradigm. Language regions are most consistently and robustly activated when participants name two objects in a picture using a phrase (e.g., “tap and pizza”) or when they name two objects in a sentence (e.g., “The cat is drinking from the jug”). By contrast, activation is much less consistent when naming a single object from a picture (single visual object naming) or naming an object from its sound (single auditory object naming). These findings have implications for pre-operative and intra-operative language mapping in that such mapping may have improved sensitivity to language function if the task involves presenting two objects in the same picture rather than pictures of single objects.

We observed greater consistency in language-related activation for naming two objects rather than one even though the number of objects presented was held constant within 25 s blocks (10 for two-object naming and 10 for single object naming). The results cannot be explained by inter-subject differences in the BOLD response *per se*, because the hemodynamic response to neural activity is expected to stay constant within region and we are comparing inter-subject variability/consistency within region. Moreover, we found no evidence that this greater consistency was the consequence of familiarity/fatigue effects. Instead, we hypothesize that naming two objects increases demand on processes related to word retrieval and production yielding robust language-related activation at the individual subject level.

In the language regions of interest used in the current study (left posterior temporal and left posterior frontal areas involved in speech processing), activation did not show significantly more consistency for producing two object names in a sentence compared to a noun phrase (see Figure 6 and Table 3). Therefore, the simpler two-object naming task was sufficient for investigating activation in our chosen language regions of interest. If, on the other hand, the regions of interest chosen were those involved in syntactic processing, the sentence production task would be a better choice for pre-surgical planning. For example, in a series of 14 neurosurgery patients, Chang et al. (2018) used DES to identify stimulation sites associated with syntactic deficits during sentence production. Stimulation of regions in the pars opercularis and pars triangularis, which have not been identified during mapping with counting, naming or repetition, induced syntactic errors in 7/14 patients.

In the temporal lobe language region of interest, which included the temporo-parietal junction, middle and superior temporal cortex, activation was more consistent for auditory object naming than for visual object naming (see Figure 6 and Table 3C). This is in line with prior studies (Hamberger et al., 2001, 2005) that reported a clinical benefit of utilizing single auditory object naming for language mapping in patients with temporal lobe epilepsy (TLE) (Hamberger et al., 2001, 2005). TLE patients often present with word finding difficulties and conversational speech impairments, despite normal performance on visual naming tasks. Carefully planned language testing, which takes into consideration the stimulus modality, is therefore crucial in preserving a patient’s quality of life, particularly since a study by Moritz-Gasser et al. (2012) demonstrated that naming ability was significantly correlated with return to work in patients with low-grade gliomas.

In addition, our results show how consistently sensory and motor regions are activated by all four object naming tasks (Figure 4). This supports the notion that object naming can be used to probe the function of many different brain regions during intra-operative mapping. An impaired response to an object naming task during DES does not, however, indicate the function of the stimulated region as so many different types of processes are involved in object naming. To determine the function of a brain region, multiple different tasks are required to systematically manipulate the demands on different types of processing. This is possible within group fMRI studies (e.g., the 16 tasks administered to Group 1) but is not feasible for pre-operative planning because single patient pre-operative fMRI mapping needs to maximize repetitions of the same task for reliable estimation of signal to noise; and this necessitates minimizing the number of tasks unless the patient can return for multiple scanning sessions. In addition, interpretation of results from multitask fMRI studies is often challenging.

Our functional consistency maps offer a potential data-based solution for pre-surgical planning, accounting for inter-subject variability. Specifically, for each region of interest, functional consistency maps can be generated to calculate inter-subject consistency in response to multiple different tasks and task differences (contrasts). Neuro-surgical teams could then compare the location of regions planned for resection with the output from a database that indicates (i) which tasks engage the region; (ii) the consistency with which the region is engaged for these tasks across neurotypical individuals; and (iii) which tasks might be optimal for pre-operative fMRI or intra-operative DES.

### Limitations and Future Directions

The current study explored the consistency of object naming activation in healthy controls. These findings may not necessarily translate to patient populations because the object naming networks may have already re-organized in patients with brain tumors or epilepsy (Fisicaro et al., 2016). To further investigate the effect of pathology on the consistency of object naming activation, future studies could investigate the consistency of language-task related activation in more heterogeneous participant samples, such as patients with brain tumors or drug-resistant epilepsy.

Our results suggest that successful implementation of the two-object naming paradigm in the intra-operative setting may allow for more sensitive language mapping. This motivates an explicit evaluation of whether the two-object naming paradigm provides a more reliable probe of language function than single object naming during intraoperative mapping. Further fMRI studies of neurotypical populations could also test how inter-subject variability/consistency changes with different inter-stimulus intervals. Based on the current results, our hypotheses are that (A) activation will be higher when participants are under time pressure but can still produce correct responses and (B) presenting two or more objects simultaneously provides a practical way to increase time pressure compared to presenting each object one at a time at a fast rate.

The approach illustrated in the current study can be extended to map networks of regions activated by different language tasks. For instance, Rofes and Miceli (2014) argued that verb naming might be more sensitive than object naming due to recruitment of additional networks involved in grammatical processing. Functional consistency maps could be used to compare the consistency of activation for verb naming relative to object naming and make further recommendations for intra-operative testing. This would contribute to data-based approaches for neurosurgical planning that will provide reliable and lesion-site specific brain mapping paradigms.

## Summary and Conclusion

Object naming is a widely utilized task in patients undergoing neurosurgery and allows the mapping of a widely distributed network of speech production regions. In this study, we examined inter-subject consistency in activation during four different object naming tasks in neurotypical participants. Naming two depicted objects either in a phrase or in a sentence resulted in more consistent activation in core language areas (posterior temporal and inferior frontal) in comparison to single object naming (from visual or auditory stimuli). We therefore propose that requiring two objects to be named on a trial may optimize sensitivity to DES effects during awake language mapping. In addition, single object naming in the auditory modality (naming object from sounds) resulted in higher consistency of activation in temporal language regions in comparison to single object naming in the visual modality (naming objects from pictures). Our findings highlight the importance of selecting a stimulus modality based on lesion site.

## Data Availability Statement

The data that support the findings of this study are available upon request from the senior author CP.

## Ethics Statement

The studies involving human participants were reviewed and approved by London Queen Square Research Ethics Committee. The patients/participants provided their written informed consent to participate in this study.

## Author Contributions

CP, JE, and MS contributed to conception and design of the study. JE performed the statistical analysis. JE and CP wrote the first draft of the manuscript. DG and MK wrote sections of the manuscript. All authors contributed to manuscript revision, read, and approved the submitted version.

## Conflict of Interest

The authors declare that the research was conducted in the absence of any commercial or financial relationships that could be construed as a potential conflict of interest.

## Publisher’s Note

All claims expressed in this article are solely those of the authors and do not necessarily represent those of their affiliated organizations, or those of the publisher, the editors and the reviewers. Any product that may be evaluated in this article, or claim that may be made by its manufacturer, is not guaranteed or endorsed by the publisher.

## References

[B1] Benjamin C. F. A. Li A. X. Blumenfeld H. Constable R. T. Alkawadri R. Bickel S. (2018). Presurgical language fMRI: clinical practices and patient outcomes in epilepsy surgical planning. *Hum. Brain Mapp.* 39 2777–2785. 10.1002/hbm.24039 29528160 PMC6033659

[B2] Berger M. S. Ojemann G. A. (1992). Intraoperative brain mapping techniques in neuro-oncology. *Stereotact. Funct. Neurosurg.* 58 153–161. 10.1159/000098989 1439333

[B3] Berger M. S. Kincaid J. Ojemann G. A. Lettich E. (1989). Brain mapping techniques to maximize resection, safety, and seizure control in children with brain tumors. *Neurosurgery* 25 786–792. 10.1097/00006123-198911000-00015 2586730

[B4] Black D. F. Vachha B. Mian A. Faro S. H. Maheshwari M. Sair H. I. (2017). American society of functional neuroradiology–recommended fMRI paradigm algorithms for presurgical language assessment. *Am. J. Neuroradiol.* 38 E65–E73. 10.3174/ajnr.A5345 28860215 PMC7963630

[B5] Bu L. Lu J. Zhang J. Wu J. (2021). Intraoperative cognitive mapping tasks for direct electrical stimulation in clinical and neuroscientific contexts. *Front. Hum. Neurosci.* 15:61. 10.3389/fnhum.2021.612891 33762913 PMC7982856

[B6] Castellano A. Cirillo S. Bello L. Riva M. Falini A. (2017). Functional MRI for surgery of gliomas. *Curr. Treat. Options Neurol.* 19 1–23. 28831723 10.1007/s11940-017-0469-y

[B7] Chang E. F. Kurteff G. Wilson S. M. (2018). Selective interference with syntactic encoding during sentence production by direct electrocortical stimulation of the inferior frontal gyrus. *J. Cogn. Neurosci.* 30 411–420. 10.1162/jocn_a_01215 29211650 PMC5819756

[B8] De Witte E. Mariën P. (2013). The neurolinguistic approach to awake surgery reviewed. *Clin. Neurol. Neurosurg.* 115 127–145. 10.1016/j.clineuro.2012.09.015 23036660

[B9] Duffau H. (2005). Lessons from brain mapping in surgery for low-grade glioma: insights into associations between tumour and brain plasticity. *Lancet Neurol.* 4 476–486. 10.1016/S1474-4422(05)70140-X 16033690

[B10] Duffau H. Moritz-Gasser S. Gatignol P. (2009). Functional outcome after language mapping for insular world health organization grade II gliomas in the dominant hemisphere: experience with 24 patients. *Neurosurg. Focus* 27:E7. 10.3171/2009.5.FOCUS0938 19645563

[B11] Duyck W. Desmet T. Verbeke L. P. C. Brysbaert M. (2004). Word gen: a tool for word selection and nonword generation in Dutch, English, German, and French. *Behav. Res. Methods, Instruments Comput.* 36 488–499. 10.3758/BF03195595 15641437

[B12] Fedorenko E. Blank I. A. (2020). Broca’s area is not a natural kind. *Trends Cogn. Sci.* 24 270–284. 10.1016/j.tics.2020.01.001 32160565 PMC7211504

[B13] Fisicaro R. A. Jost E. Shaw K. Brennan N. P. Peck K. K. Holodny A. I. (2016). Cortical plasticity in the setting of brain tumors. *Top. Magn. Reson. Imaging TMRI* 25:25. 10.1097/rmr.0000000000000077 26848558 PMC4970642

[B14] Friston K. J. Frith C. D. Turner R. Frackowiak R. S. J. (1995). Characterizing evoked hemodynamics with fMRI. *Neuroimage* 2 157–165. 10.1006/nimg.1995.1018 9343598

[B15] Gabel N. Altshuler D. B. Brezzell A. Briceno E. M. Boileau N. R. Miklja Z. (2019). Health related quality of life in adult low and high-grade glioma patients using the national institutes of health patient reported outcomes measurement information system (PROMIS) and neuro-QOL assessments. *Front. Neurol.* 10:212. 10.3389/fneur.2019.00212 30930834 PMC6428723

[B16] Giussani C. Roux F.-E. Ojemann J. Sganzerla E. Pirillo D. Papagno C. (2010). Is preoperative functional magnetic resonance imaging reliable for language areas mapping in brain tumor surgery? Review of language functional magnetic resonance imaging and direct cortical stimulation correlation studies. *Neurosurgery* 66 113–120. 10.1227/01.NEU.0000360392.15450.C9 19935438

[B17] Hamberger M. J. Goodman R. R. Perrine K. Tamny T. (2001). Anatomic dissociation of auditory and visual naming in the lateral temporal cortex. *Neurology* 56 56–61. 10.1212/wnl.56.1.56 11148236

[B18] Hamberger M. J. Seidel W. T. Mckhann G. M. Perrine K. Goodman R. R. (2005). Brain stimulation reveals critical auditory naming cortex. *Brain* 128 2742–2749. 10.1093/brain/awh621 16109751

[B19] Hamer P. C. D. W. Robles S. G. Zwinderman A. H. Duffau H. Berger M. S. (2012). Impact of intraoperative stimulation brain mapping on glioma surgery outcome: a meta-analysis. *J. Clin. Oncol.* 30 2559–2565. 10.1200/JCO.2011.38.4818 22529254

[B20] Hervey-Jumper S. L. Li J. Lau D. Molinaro A. M. Perry D. W. Meng L. (2015). Awake craniotomy to maximize glioma resection: methods and technical nuances over a 27-year period. *J. Neurosurg.* 123 325–339. 10.3171/2014.10.JNS141520 25909573

[B21] Hocking J. Dzafic I. Kazovsky M. Copland D. A. (2013). NESSTI: norms for environmental sound stimuli. *PLoS One* 8:e73382. 10.1371/journal.pone.0073382 24023866 PMC3762767

[B22] Hope T. M. H. Prejawa S. Parker Jones O. Oberhuber M. Seghier M. L. Green D. W. (2014). Dissecting the functional anatomy of auditory word repetition. *Front. Hum. Neurosci.* 8:1–17. 10.3389/fnhum.2014.00246 24834043 PMC4018561

[B23] Ilmberger J. Ruge M. Kreth F.-W. Briegel J. Reulen H.-J. Tonn J.-C. (2008). Intraoperative mapping of language functions: a longitudinal neurolinguistic analysis. *J. Neurosurg.* 109 583–592. 10.3171/JNS/2008/109/10/0583 18826344

[B24] Ius T. Isola M. Budai R. Pauletto G. Tomasino B. Fadiga L. (2012). Low-grade glioma surgery in eloquent areas: volumetric analysis of extent of resection and its impact on overall survival. a single-institution experience in 190 patients. *J. Neurosurg.* 117 1039–1052. 10.3171/2012.8.jns12393 23039150

[B25] Jakola A. S. Myrmel K. S. Kloster R. Torp S. H. Lindal S. Unsgård G. (2012). Comparison of a strategy favoring early surgical resection vs a strategy favoring watchful waiting in low-grade gliomas. *JAMA J. Am. Med. Assoc.* 308 1881–1888. 10.1001/jama.2012.12807 23099483

[B26] Jakola A. S. Skjulsvik A. J. Myrmel K. S. Sjåvik K. Unsgård G. Torp S. H. (2017). Surgical resection versus watchful waiting in low-grade gliomas. *Ann. Oncol.* 28 1942–1948. 10.1093/annonc/mdx230 28475680 PMC5834105

[B27] Jakola A. S. Unsgård G. Solheim O. (2011). Quality of life in patients with intracranial gliomas: the impact of modern image-guided surgery. *J. Neurosurg.* 114 1622–1630. 10.3171/2011.1.JNS101657 21314270

[B28] Klitsinikos D. Ekert J. O. Carels A. Samandouras G. (2021). Mapping and anatomo-surgical techniques for SMA-cingulum-corpus callosum gliomas; how i do it. *Acta Neurochir. (Wien)* 163 1239–1246. 10.1007/s00701-021-04774-7 33779836 PMC8053665

[B29] Leon-Rojas J. E. Ekert J. O. Kirkman M. A. Sewell D. Bisdas S. Samandouras G. (2020). Experience with awake throughout craniotomy in tumour surgery: technique and outcomes of a prospective, consecutive case series with patient perception data. *Acta Neurochir. (Wien)* 162 3055–3065. 10.1007/s00701-020-04561-w 33006649

[B30] Mahdavi A. Azar R. Shoar M. H. Hooshmand S. Mahdavi A. Kharrazi H. H. (2015). Functional MRI in clinical practice: assessment of language and motor for pre-surgical planning. *Neuroradiol. J.* 28 468–473. 10.1177/1971400915609343 26443298 PMC4757221

[B31] Manan H. A. Franz E. A. Yahya N. (2020). Utilization of functional MRI language paradigms for pre-operative mapping: a systematic review. *Neuroradiology* 62 353–367. 10.1007/s00234-019-02322-w 31802156

[B32] Mandonnet E. Winkler P. A. Duffau H. (2010). Direct electrical stimulation as an input gate into brain functional networks: principles, advantages and limitations. *Acta Neurochir. (Wien)* 152 185–193. 10.1007/s00701-009-0469-0 19639247

[B33] Matsumoto R. Nair D. R. LaPresto E. Najm I. Bingaman W. Shibasaki H. (2004). Functional connectivity in the human language system: a cortico-cortical evoked potential study. *Brain* 127 2316–2330. 10.1093/brain/awh246 15269116

[B34] McGirt M. J. Chaichana K. L. Gathinji M. Attenello F. J. Than K. Olivi A. (2009). Independent association of extent of resection with survival in patients with malignant brain astrocytoma. *J. Neurosurg.* 110 156–162. 18847342 10.3171/2008.4.17536

[B35] Moritz-Gasser S. Herbet G. Maldonado I. L. Duffau H. (2012). Lexical access speed is significantly correlated with the return to professional activities after awake surgery for low-grade gliomas. *J. Neurooncol.* 107 633–641. 10.1007/s11060-011-0789-9 22270847

[B36] O’neill M. Henderson M. Duffy O. M. Kernohan W. G. (2020). The emerging contribution of speech and language therapists in awake craniotomy: a national survey of their roles, practices and perceptions. *Int. J. Lang. Commun. Disord.* 55 149–162. 10.1111/1460-6984.12510 31778003

[B37] Ojemann G. A. Ojemann J. G. Lettich E. Berger M. (1989). Cortical language localization in left, dominant hemisphere: an electrical stimulation mapping investigation in 117 patients. *J. Neurosurg.* 71 316–326. 2769383 10.3171/jns.1989.71.3.0316

[B38] Oldfield R. C. (1971). The assessment and analysis of handedness: the Edinburgh inventory. *Neuropsychologia* 9 97–113. 10.1016/0028-3932(71)90067-4 5146491

[B39] Pak R. W. Hadjiabadi D. H. Senarathna J. Agarwal S. Thakor N. V. Pillai J. J. (2017). Implications of neurovascular uncoupling in functional magnetic resonance imaging (fMRI) of brain tumors. *J. Cereb. Blood Flow Metab.* 37 3475–3487. 10.1177/0271678X17707398 28492341 PMC5669348

[B40] Rofes A. Miceli G. (2014). Language mapping with verbs and sentences in awake surgery: a review. *Neuropsychol. Rev.* 24 185–199. 10.1007/s11065-014-9258-5 24736866

[B41] Rofes A. de Aguiar V. Miceli G. (2015). A minimal standardization setting for language mapping tests: an Italian example. *Neurol. Sci.* 36 1113–1119. 10.1007/s10072-015-2192-3 25851729

[B42] Rofes A. Spena G. Talacchi A. Santini B. Miozzo A. Miceli G. (2017b). Mapping nouns and finite verbs in left hemisphere tumors: a direct electrical stimulation study. *Neurocase* 23 105–113. 10.1080/13554794.2017.1307418 28347212

[B43] Rofes A. Mandonnet E. Godden J. Baron M. H. Colle H. Darlix A. (2017a). Survey on current cognitive practices within the European low-grade glioma network: towards a European assessment protocol. *Acta Neurochir. (Wien)* 159 1167–1178. 10.1007/s00701-017-3192-2 28474122

[B44] Ruis C. (2018). Monitoring cognition during awake brain surgery in adults: a systematic review. *J. Clin. Exp. Neuropsychol.* 40 1081–1104. 10.1080/13803395.2018.1469602 30067443

[B45] Sanai N. Berger M. S. (2008a). Glioma extent of resection and its impact on patient outcome. *Neurosurgery* 62 753–766. 10.1227/01.neu.0000318159.21731.cf 18496181

[B46] Sanai N. Berger M. S. (2008b). Mapping the horizon: techniques to optimize tumor resection before and during. *Clin. Neurosurg.* 55:14. 19248664

[B47] Sanai N. Berger M. S. (2010). Intraoperative stimulation techniques for functional pathway preservation and glioma resection. *Neurosurg. Focus* 28:E1. 10.3171/2009.12.FOCUS09266 20121436

[B48] Sanai N. Mirzadeh Z. Berger M. S. (2008). Functional outcome after language mapping for glioma resection. *N. Engl. J. Med.* 358 18–27. 10.1056/nejmoa067819 18172171

[B49] Sanjuán A. Hope T. M. H. Parker Jones O. Prejawa S. Oberhuber M. Guerin J. (2015). Dissociating the semantic function of two neighbouring subregions in the left lateral anterior temporal lobe. *Neuropsychologia* 76 153–162. 10.1016/j.neuropsychologia.2014.12.004 25496810 PMC4582806

[B50] Sefcikova V. Sporrer J. K. Ekert J. O. Kirkman M. A. Samandouras G. (2020). High interrater variability in intraoperative language testing and interpretation in awake brain mapping among neurosurgeons or neuropsychologists: an emerging need for standardization. *World Neurosurg.* 141 e651–e660. 10.1016/j.wneu.2020.05.250 32522656

[B51] Seghier M. L. Price C. J. (2016). Visualising inter-subject variability in fMRI using threshold-weighted overlap maps. *Sci. Rep.* 6:20170. 10.1038/srep20170 26846561 PMC4742862

[B52] Seghier M. L. Price C. J. (2018). Interpreting and utilising intersubject variability in brain function. *Trends Cogn. Sci.* 22 517–530. 10.1016/j.tics.2018.03.003 29609894 PMC5962820

[B53] Seghier M. L. Patel E. Prejawa S. Ramsden S. Selmer A. Lim L. (2016). The PLORAS database: a data repository for predicting language outcome and recovery after stroke. *Neuroimage* 124 1208–1212. 10.1016/j.neuroimage.2015.03.083 25882753 PMC4658335

[B54] Shimotake A. Matsumoto R. Ueno T. Kunieda T. Saito S. Hoffman P. (2015). Direct exploration of the role of the ventral anterior temporal lobe in semantic memory: cortical stimulation and local field potential evidence from subdural grid electrodes. *Cereb. Cortex* 25 3802–3817. 10.1093/cercor/bhu262 25491206 PMC4585516

[B55] Silva M. A. See A. P. Essayed W. I. Golby A. J. Tie Y. (2018). Challenges and techniques for presurgical brain mapping with functional MRI. *NeuroImage Clin.* 17 794–803. 10.1016/j.nicl.2017.12.008 29270359 PMC5735325

[B56] Unadkat P. Fumagalli L. Rigolo L. Vangel M. G. Young G. S. Huang R. (2019). Functional MRI task comparison for language mapping in neurosurgical patients. *J. Neuroimaging* 29 348–356. 10.1111/jon.12597 30648771 PMC6506353

[B57] Van Turennout M. Bielamowicz L. Martin A. (2003). Modulation of neural activity during object naming: effects of time and practice. *Cereb. Cortex* 13 381–391. 10.1093/cercor/13.4.381 12631567

[B58] Young J. S. Lee A. T. Chang E. F. (2021). A review of cortical and subcortical stimulation mapping for language. *Neurosurgery* 89 331–342. 33444451 10.1093/neuros/nyaa436 PMC8492609

